# Safety of three different product doses in autologous chondrocyte implantation: results of a prospective, randomised, controlled trial

**DOI:** 10.1186/s13018-017-0570-7

**Published:** 2017-05-12

**Authors:** Christoph Becher, Volker Laute, Stefan Fickert, Wolfgang Zinser, Philipp Niemeyer, Thilo John, Peter Diehl, Thomas Kolombe, Rainer Siebold, Jakob Fay

**Affiliations:** 1 Department of Orthopedic Surgery, Hannover Medical School, Anna-von-Borries-Str. 1-7, 30625 Hannover, Germany; 2 Joint and Spine Centre Berlin, Berlin, Germany; 3 Sporthopaedicum Straubing, Straubing, Germany; 4 Department of Orthopedic Surgery and Traumatology, St. Vinzenz-Hospital, Dinslaken, Germany; 5 Department of Orthopedic Surgery and Traumatology, Freiburg University Hospital, Freiburg, Germany; 6 Clinic for Traumatology and Orthopedic Surgery, DRK Hospital Westend, Berlin, Germany; 7 Department of Orthopedic Surgery and Traumatology, Orthopedic Center Munich East, München, Germany; 8 Traumatology and Reconstructive Surgery, DRK Hospital, Luckenwalde, Germany; 9 Center for Hip, Knee and Foot Surgery, ATOS Clinic, Heidelberg, Germany; 10 Department of Traumatology and Arthroscopic Surgery, Lubinus Clinicum, Kiel, Germany

**Keywords:** Knee, ACI, Cartilage, Safety, Prospective randomised trial

## Abstract

**Background:**

This study was conducted to assess the efficacy and safety of the three dose levels of the three-dimensional autologous chondrocyte implantation product chondrosphere® in the treatment of cartilage defects (4–10 cm^2^) of knee joints. We hereby report the safety results for a 36-month post-treatment observation period.

**Methods:**

This was a prospective phase II trial with a clinical intervention comprising biopsy for culturing spheroids and their subsequent administration (level of evidence: I). Patients’ knee defects were investigated by arthroscopy, and a cartilage biopsy was taken for culturing. Patients were randomised, on a single-blind basis, to treatment at the dose levels 3–7 (low), 10–30 (medium) or 40–70 (high) spheroids per square centimetre. Assessment (adverse events, vital signs, electrocardiography, physical examination, concomitant medication and laboratory values) took place 1.5, 3, 6, 12, 24 and 36 months after chondrocyte implantation.

**Results:**

Seventy-five patients were included and 73 treated. The incidence of adverse events, of patients with adverse events and of patients with treatment-related adverse events showed no relevant difference between the treatment groups. There were no fatal adverse events, no adverse events led to premature withdrawal from the trial and none led to permanent sequelae. Two patients experienced serious adverse events considered related to the study treatment: arthralgia 2–3 years after implantation and chondropathy 1 and 2 years after implantation.

**Conclusions:**

The treatment with chondrosphere® was generally well tolerated. No relationship was detected between any safety criteria and the dose level: Differences between the dose groups in the incidence of any adverse events, and in numbers of patients with treatment-related adverse events, were insubstantial.

**Trial registration:**

clinicaltrials.gov, NCT01225575.

## Background

Articular cartilage injury is a common orthopaedic problem, and repair of articular cartilage defects remains one of the most challenging problems in orthopaedic surgery. The capacity of articular cartilage for self-repair is limited, and experience with repair techniques such as drilling and microfracture have affirmed the difficulty of providing durable function and relief of pain and preventing progression to osteoarthritis [1, 2]. Autologous chondrocyte implantation (ACI) results in more hyaline-like cartilage with biomechanical characteristics better than those obtained by microfracture or drilling [3]. The procedure has been in clinical use since 1987 and was based on the implantation of a suspension of cultured autologous chondrocytes beneath a tightly sealed periosteal flap [4]. The classical ACI technique has been modified over the years and has led to a new generations of cell-based cartilage repair procedures, with the use of three-dimensional matrix scaffolds that facilitate the procedure and show favourable biological properties [5].

A further development of matrix-based techniques is the application of chondrocytes as spheroids. The mode of action of ACI with chondrocyte spheroids (chondrosphere®, ACT3D-CS, co.don AG, Teltow, Germany) is based on the integration of implanted spheroids into the cartilage defect, mediated by chondrocyte migration and synthesis de novo of matrix elements of hyaline-like cartilage. To date, no well-controlled clinical trials have been reported that compared various doses of cells per square centimetre in ACI. Likewise, no studies have yet been performed to compare the effectiveness of different numbers of cells for a given defect size. In general, commercially available sources of chondrocyte suspensions recommend a dose between 0.5–1.0 and 2.0–3.0 × 10^6^ cells per square centimetre [3, 6]. In a study conducted for approval of an autologous cell product by the European Medicines Agency (EMA), cell densities in this range were also used [7, 8]. However, there is still a debate as to whether an increased cell density can lead to better cartilage filling, better morphological structure biomechanical properties and/or a more favourable clinical outcome [9, 10]. Thus, there remains a crucial need for studies evaluating the efficacy and safety of different cell densities and product doses. The autologous chondrocyte implant chondrosphere® has been marketed, and has been licensed in Germany, since 2004. Chondrocyte spheroids (chondrosphere®) are generated by seeding 2 × 10^5^ chondrocytes in a three-dimensional cell cultivation system.

The standard chondrosphere® treatment in clinical practice is 10–70 spheroids/cm^2^, and this study was designed to compare three dose levels: two within the standard range (10–30 spheroids/cm^2^ and 40–70 spheroids/cm^2^) and one below it (3–7 spheroids/cm^2^) in order to establish a minimum effective dose and to assess and compare the safety of all three doses. The initial efficacy of the product in terms of MRI morphological evaluation of repair tissue was recently published [11]. Furthermore, according to the ICRS-CRA (International Cartilage Repair Score, Cartilage Repair Assessment) and KOOS (Knee Injury and Osteoarthritis Outcome Score), good second-look arthroscopy and clinical results were found in a single-surgeon cohort study [12]. The objective of this paper is to present the safety results and thus to assess the safety of the three dose levels of the three-dimensional autologous chondrocyte implantation product ACT3D-CS in the treatment of cartilage defects (4–10 cm^2^) of knee joints over a 36-month observation period.

## Methods

### Study design

This was a phase II, randomised, prospective trial with a clinical intervention comprising biopsy for culturing spheroids and subsequent administration at the dose levels stated above. For ethical reasons, no placebo control was used, and since no alternative treatment is known to be effective for large cartilage defects, there was also no active comparator. Patients were blinded to their dose level; physicians were not (single-blind design). The design of this clinical study and its conduct (at ten orthopaedic clinics in Germany) met all legal and regulatory requirements and were compliant with the Good Clinical Practice and the Declaration of Helsinki. The study was approved by the relevant ethics committees. All patients gave their written informed consent to participate.

### Treatment and assessments

The study therapy comprised implantation of ACI spheroids (chondrosphere*®*), cultured from samples taken from the patient (biopsy from the affected joint and serum), into the damaged cartilage region.

Patients’ knee defects were investigated by arthroscopy, and a cartilage biopsy was taken; the date of the examination immediately preceding the arthroscopy is termed day 0. Cells from the biopsy were cultured to yield chondrocytes (chondrosphere®). In a second arthroscopy 6–7 weeks after day 0, the defect was debrided and chondrocytes were implanted according to the manufacturer’s instructions; the date of the examination immediately preceding this is termed day 0. After surgery, patients underwent a standard rehabilitation programme that started during their stay in the clinic and continued at home for up to 3 months after the intervention.

Patients returned to the treatment centre for assessment after 6 weeks and 3, 6 and 12 months after chondrocyte implantation, and for follow-up after 24 and 36 months; further follow-up is planned for 48 and 60 months. For all the results shown here, the cut-off date was 36 months after the implantation procedure.

### Patients

Since ACI is regarded as the best treatment option and current therapeutic standard for medium to large defects (larger than 3–4 cm^2^), only patients with defect size of 4–10 cm^2^ were included. Other principal inclusion criteria were age 18–50 years; isolated ICRS grade III or IV single defect on medial or lateral femoral condyle, trochlea, tibia and retropatellar defect, also osteochondritis dissecans (for osteochondritis dissecans, bone grafting up to the level of the original bone lamella was to be performed if bone loss exceeded 3 mm in depth); nearly intact chondral structure surrounding the defect and corresponding joint area; certain restrictions on pain medication, especially immediately before study visits; agreement to participate fully in the rehabilitation programme (see below). Principal exclusion criteria were bilateral defects or two defects in the same knee; radiological signs of osteoarthritis; knee instability; valgus or varus misalignment >5°; 50% resection of a meniscus in the affected knee or incomplete meniscal rim; rheumatoid, parainfectious or infectious arthritis; obesity (body mass index > 30 kg/m^2^; meniscal implant or recent suture in the affected knee; other criteria designed to avoid jeopardising the patient or the study result. Patients were stratified prospectively after the biopsy-taking and the end of arthroscopy, according to defect size (≥4–<7 cm^2^ and ≥7–10 cm^2^), and within each defect size group, they were allocated by central randomisation (1:1:1) to treatment with 3–7 spheroids/cm^2^, 10–30 spheroids/cm^2^ or 40–70 spheroids/cm^2^.

### Safety evaluation criteria and analysis

Safety criteria were adverse events, vital signs including electrocardiography, physical examination, concomitant pain medication and laboratory values. Safety analysis was carried out, using the software SAS 9.2 for Microsoft Windows (SAS, Cary NC, USA), by tabulation of adverse events (numbers of reports and numbers/percentages of patients affected) and by presenting descriptive statistics for continuous variables.

## Results

A total of 163 patients with unilateral knee defects were screened between November 2010 and September 2012. In 43 cases, the defect was too small for inclusion in the study and another 45 failed to meet other eligibility criteria. The remaining 75 patients were included in the study, underwent biopsy and were randomised for low-, medium- or high-dose treatment (25 patients each). For two patients, randomised respectively to the low-dose and high-dose groups, the chondrocytes did not grow, making implantation impossible, but as these patients had been randomised and had undergone an invasive procedure, they were included in the population for safety analysis.

Deviations from the study protocol affected two patients who, after arthroscopy and debridement, were found not to have satisfied the inclusion criteria: one (low-dose group) had a defect size below 2 cm^2^, and the other (high-dose group) had two defects in the affected knee. Other potentially safety-related deviations (one patient each) were use of a membrane (high-dose group), use of a lateral meniscal suture (high-dose group), failure to perform bone grafting (medium-dose group) and performance of the implantation by a physician who was not an investigator in the study (low-dose group). Other deviations from protocol were not considered safety-relevant.

Knee defects, as determined on the day of arthroscopy, affected the femur in 28/75 cases and the patella in 47/75 cases. According to the ICRS classification [5], defects were mostly of grade IV A (in 43 cases) or III C (in 16 cases).

Apart from the study indication, the patients’ medical histories were inconspicuous, and none were considered likely to influence the study result. Demographic information and details of the patients’ baseline condition are given in Table 1.

**Table 1:** Study patients: demography and baseline characteristics (all patients)

Dose group Dose [spheroids/cm^2^]	Low	Medium	High	All patients
	3–7	10–30	40–70	
	*N* = 25	*N* = 25	*N* = 25	*N* = 75
Sex				
Female	8	4	10	22
Male	17	21	15	53
Age [years]	33 ± 10	34 ± 9	34 ± 9	34 ± 9
BMI				
[kg/m^2^]	24.9 ± 2.5	25.6 ± 3.2	25.1 ± 3.6	25.2 ± 3.1
Range	21.3–29.8	19.4–33.2	19.0–32.3	19.0–33.2
Defect size				
[cm^2^]	4.8 ± 1.5	4.9 ± 1.3	5.2 ± 1.3	5.0 ± 1.9
Range	0.5^a^–7.5	1.3^a^–7.5	3.0^a^–8.0	0.5^a^–8.0
Defect size group				
4–6.99 cm^2^	22	22	21	65
7–10 cm^2^	3	3	4	10
Defect location (primary)				
Femur	9	10	9	28
Tibia	–	–	–	–
Patella	16	15	16	47

Numbers of patients or mean ± SD, or where appropriate the range (minimum–maximum), are given

^a^Value outside allowed range

Exposure to the test product is summarised in Table 2. The differences between the doses per square centimetre at arthroscopy and at implantation are due to the difficulty in assessing the size of the defect by arthroscopy before debridement. Following the surgery, 72 patients completed the rehabilitation programme; one withdrew from the study directly after implantation.

**Table 2:** Exposure to the test product: dose administered

Dose group:	Low	Medium	High	All
	*N* = 24	*N* = 25	*N* = 24	*N* = 73
Spheroids/cm^2^ based on defect area as found by arthroscopy^a^				
Mean ± SD	10.8 ± 15.7	27.8 ± 13.0	40.5 ± 11.0	26.44 ± 17.9
Median	7.0	28.8	41.80	28.2
Range	6.8–84.0	11.3–83.1	11.6–59.8	6.8–84.0
Spheroids/cm^2^ based on defect area as found at implantation^a^				
Mean ± SD	7.6 ± 3.1	23.3 ± 6.7	37.7 ± 12.4	22.8 ± 14.8
Median	7.0	24.7	40.7	23.4
Range	4.7–21.0	9.3–30.7	11.6–59.8	4.7–59.8
Number of spheroids				
Mean ± SD	37.5 ± 10.8	128.1 ± 41.3	204.3 ± 51.4	123.3 ± 78.1
Median	35	120	209	120
Range	28–63	51–224	93–290	28–290

^a^Area at arthroscopy was used for determination of dose (amount of chondrosphere®); area at implantation was post-debridement and therefore more accurate (see text)

### Adverse events

In the 3-year period covered by this report, there were 79 reports of adverse events in the low-dose group, 71 such reports in the medium-dose group and 112 in the high-dose group (262 in total). There were no fatal adverse events, no adverse events led to premature withdrawal from the trial, and none led to permanent sequelae; however, 40 adverse events had not yet been resolved by the cut-off date for analysis, and there were two adverse events with unknown outcome.

A complete summary of adverse events in the 3-year period is shown in Table 3 and of those considered (at least possibly) related to the study treatment in Table 4. The overall incidence of adverse events, of patients with any adverse events and of patients with treatment-related adverse events in the 36-month assessment did not differ conspicuously between the treatment groups, and no dose dependence was found.

**Table 3:** Adverse events (safety population)

Dose group	Low		Medium		High		All	
	*N* = 25		*N* = 25		*N* = 25		*N* = 75	
	*n*_P_	*n*_E_	*n*_P_	*n*_E_	*n*_P_	*n*_E_	*n*_P_	*n*_E_
Any SOC	23	79	24	71	22	112	69	262
Musculoskeletal and connective tissue disorders	18	41	24	44	22	48	64	133
Joint effusion	17	20	22	27	20	27	59	74
Arthralgia	6	10	4	4	6	8	16	22
Joint swelling	3	4	2	3	1	2	6	9
Joint crepitation	–	–	4	4	2	2	6	6
Chondropathy	1	1	1	1	1	2	3	4
Back pain	1	1	–	–	2	2	3	3
Tendonitis	–	–	1	1	2	2	3	3
Joint lock	–	–	2	2	–	–	2	2
Muscular weakness	1	2	–	–	–	–	1	2
Nervous system disorders	5	16	2	2	1	30	8	48
Infections and infestations	5	7	8	10	4	7	17	24
Injury, poisoning and procedural complications	6	6	5	7	7	10	18	23
Gastrointestinal disorders	1	1	1	1	3	5	5	7
Metabolism and nutrition disorders	1	1	–	–	2	4	3	5
General disorders and admin. site conditions	1	1	1	2	1	1	3	4
Vascular disorders	1	1	1	1	2	2	4	4
Cardiac disorders	1	1	1	1	–	–	2	2
Ear and labyrinth disorders	1	1	–	–	1	1	2	2
Immune system disorders	1	1	1	1	–	–	2	2
Surgical and medical procedures	–	–	–	–	1	2	1	2

MedDRA SOC and preferred terms are used. Numbers of patients (*n*_P_) and events (*n*_E_) are given. Inclusion for all *n*_P_ (all) >1

**Table 4:** Adverse events considered probably or possibly treatment-related (safety population)

Dose group	Low		Medium		High		All	
	*N* = 25		*N* = 25		*N* = 25		*N* = 75	
	*n*_P_	*n*_E_	*n*_P_	*n*_E_	*n*_P_	*n*_E_	*n*_P_	*n*_E_
Any SOC	17	34	24	41	22	38	63	113
Musculoskeletal and connective tissue disorders	17	30	24	37	22	34	63	101
Joint effusion	17	20	22	27	19	25	58	72
Arthralgia	3	4	2	2	3	4	8	10
Joint swelling	2	3	2	2	–	–	4	5
Joint crepitation	–	–	3	3	2	2	5	5
Chondropathy	–	–	–	–	1	2	1	2
Muscular weakness	1	2	–	–	–	–	1	2
Joint lock	–	–	2	2	–	–	2	2
Muscle atrophy	–	–	1	1	–	–	1	1
Patellofemoral pain syndrome	1	1	–	–	–	–	1	1
Tendonitis	–	–	–	–	1	1	1	1
Injury, poisoning and procedural complications	–	–	3	3	3	3	6	6
Ligament sprain	–	–	–	–	3	3	3	3
Fall	–	–	2	2	–	–	2	2
Wound dehiscence	–	–	1	1	–	–	1	1
General disorders and admin. site conditions	1	1	1	1	–	–	2	2
Pain	1	1	1	1	–	–	2	2
Vascular disorders	1	1	–	–	1	1	2	2
Deep vein thrombosis	–	–	–	–	1	1	1	1
Lymphoedema	1	1	–	–	–	–	1	1
Cardiac disorders	1	1	–	–	–	–	1	1
Tachycardia	1	1	–	–	–	–	1	1
Ear and labyrinth disorders	1	1	–	–	–	–	1	1
Sudden hearing loss	1	1	–	–	–	–	1	1

Numbers of patients (*n*_P_) and events (*n*_E_) are given

In view of the study indication, adverse events in the class ‘musculoskeletal and connective tissue disorders’ are of particular interest. These are tabulated in full in Table 5. Numbers of reports in the three dose groups were 41 (low), 44 (medium) and 48 (high). The number of patients with ‘possibly’ and/or ‘probably’ treatment-related adverse events in this organ class was smaller in the high-dose group (22 patients, 88%; 34 events) than in the medium-dose group (24 patients, 96%; 41 events). Although this small difference cannot be regarded as clinically or statistically relevant, it at least shows that there was no suggestion of any noteworthy increase in numbers of adverse events or patients affected in the high-dose group vis-à-vis the medium-dose group.

**Table 5:** Adverse events in the SOC ‘musculoskeletal and connective tissue disorders’ (safety population)

Dose group	Low dose				Medium dose				High dose			
	*N* = 25				*N* = 25				*N* = 25			
Relationship	NR	Unl	Pos	Pro	NR	Unl	Pos	Pro	NR	Unl	Pos	Pro
Any adverse event	3	5	6	14	3	3	7	23	6	4	7	19
Joint effusion	–	–	4	13	–	–	2	21	1	1	2	17
Arthralgia	2	2	2	1	–	2	1	1	3	1	3	1
Joint swelling	–	1	–	2	1	–	–	2	1	1	–	–
Joint crepitation	–	–	–	–	1	–	2	1	–	–	1	1
Chondropathy	–	1	–	–	1	–	–	–	–	–	–	2
Back pain	–	1	–	–	–	–	–	–	2	–	–	–
Tendonitis	–	–	–	–	–	1	–	–	–	1	1	–
Joint lock	–	–	–	–	–	–	2	–	–	–	–	–
Muscular weakness	–	–	1	–	–	–	–	–	–	–	–	–
Bone cyst	1	–	–	–	–	–	–	–	–	–	–	–
Bone pain	–	1	–	–	–	–	–	–	1	–	–	–
Intervertebral disc prot.	–	–	–	–	1	–	–	–	–	–	–	–
Ligament disorder	–	1	–	–	–	–	–	–	–	–	–	–
Muscle atrophy	–	–	–	–	–	–	–	1	–	–	–	–
Myalgia	–	–	–	–	–	–	–	–	–	1	–	–
Osteoarthritis	–	–	–	–	–	–	–	–	1	–	–	–
Patellofemoral pain sy.	–	–	1	–	–	–	–	–	–	–	–	–

Numbers of patients are given

*prot.* protrusion, *sy.* syndrome

The system organ class (SOC) most often affected was the ‘musculoskeletal and connective tissue disorders’; in view of the procedure carried out, this is to be expected. Adverse events in other SOCs were less frequent; in fact, most occurred once only. It is noteworthy that there were only two reports of ‘immune system disorders’ (drug hypersensitivity in the low-dose group and house dust allergy in the medium-dose group). ‘Infections and infestations’ affected more patients in the medium-dose group, and this may have been related to a higher frequency of such ailments in this treatment group at screening.

Other conceivably treatment-related events were ‘ligament sprain’, ‘fall’ and ‘meniscus lesion’. The eight reports of ‘ligament sprain’ and their respective assessed relationships to the study treatment were as follows: 4 unrelated, 1 unlikely, 1 possible, 2 probable. The two cases of ‘fall’ (patients nos. 1109 and 1207) were rated as possibly treatment-related. The two cases each of rib fracture and meniscus lesion were treatment-unrelated. Other adverse events were mainly common ailments: headache, nasopharyngitis, influenza and diarrhoea. Numbers for ‘headache’ were increased substantially in the high-dose group because of a single subject with numerous reports of this.

Severe adverse events affected 1, 2 and 3 patients in the low-, medium- and high-dose groups respectively. Adverse events graded as ‘moderate’ affected 16, 12 and 16 patients, respectively, and events graded as ‘mild’ 21, 22 and 17 patients. The severe adverse events were meniscus lesion (low dose; unrelated to the study treatment), syncope (medium dose; unrelated), joint effusion (medium dose; probably related), arthralgia (high dose; possibly related), joint effusion (high dose; probably related) and chondropathy (high dose; two episodes, both probably related).

### Serious adverse events

There were 12 reports of serious adverse events, affecting 11 patients, as shown in Table 6. Three of these events were graded as severe. Two reoperations were recorded, one because of a meniscus lesion that was considered unrelated to the study treatment.

**Table 6:** Serious adverse events (safety population)

Dose group	Adverse event	During year after implantation	Severity	Relationship to treatment	Outcome
Low	Convulsion	1st	Moderate	None	Resolved
	Arthralgia	1st	Moderate	None	Resolved
	Meniscus lesion	2nd	Severe	None	Resolved
	Chondropathy	3rd	Moderate	Unlikely	Resolved
	Arthralgia	3rd	Moderate	Probable	Resolved
	Uterine cyst	3rd	Moderate	None	Resolved
Medium	Syncope	2nd	Severe	None	Resolved
	Chondropathy	3rd	Moderate	None	Not resolved
High	Umbilical hernia	2nd	Mild	None	Resolved
	Chondropathy^a^	2nd	Severe	Probable	Resolved
	Arthralgia	3rd	Moderate	None	Resolved

^a^Two episodes in the same patient

Two serious adverse events were considered by the investigator to have been probably (and none as ‘possibly’) related to the study treatment. These two events were arthralgia suffered by one patient 2–3 years after implantation and two separate episodes of chondropathy suffered by another patient (in the year after implantation and again approximately 2 years later). For the former patient, MRI 24 months after ACI showed mainly transplant hypertrophy (trochlear) that was an indication for surgery. The hypertrophy found was in the trochlea, while the initial defect that had received study treatment had been located in the patella. Surgery (arthroscopy and resection of hypertrophied tissue) was performed 27 months after ACI, with resolution of the adverse event recorded on the same day. The investigator considered that the serious adverse event was probably related to the study treatment and the sponsor that the event was possibly related to it.

For the patient with chondropathy, the patient suffered from a bone oedema (femur condyle, left knee) 14 months after ACI. This was resolved after treatment with a bisphosphonate. A subchondral cyst and a hint of partial posterior delamination (left knee) with subchondral bone necrosis was found later and treated with osteochondral transplantation 21 months after ACI. According to the surgery report, the regenerated cartilage in the area of the medial femur condyle was found to be very good. In the posterior parts of the femur condyle, delaminated cartilage tissue was found and removed. The subchondral bone was clearly affected (broken subchondral bone with destroyed lamella), which confirmed the indication for osteochondral transplantation. The first episode was considered by the investigator and sponsor to be probably treatment-related; the investigator also regarded the second episode as treatment-related, while the sponsor considered that the surrounding circumstances (fall, knee distortion, preexisting bone marrow oedema at inclusion) were the factors that caused the need for the repeated operation, without excluding a possible causal relationship with the study treatment.

### Other safety assessments

Laboratory measurements were conducted up to 12 months after implantation. They comprised complete blood count, hepatic tests (aspartate and alanine transaminases, γ-glutamyl transferase and bilirubin) and metabolic tests (cholesterol and triglycerides). Analysis by using scatter plots and shift tables did not reveal any tendency towards a general migration of values. None of these results gave rise to any clinical concern about the safety of the study treatment. Routine pregnancy tests gave no positive results. Laboratory-related adverse events were mild hypothyroidism for patient in the low-dose group (6 months after the patient’s implantation operation) and vitamin D deficiency for another in the high-dose group (11 months after implantation). Both were considered by the investigator to be unrelated to the study treatment.

Systolic blood pressure, diastolic blood pressure, pulse rate, body weight and body mass index were measured at all study visits; in addition, body temperature was measured and electrocardiography was performed before arthroscopy, before implantation and at the 12-month examination. None of these results revealed any noteworthy changes, and none gave rise to any clinical concern about the safety of the study treatment.

The physical examinations and the analysis of concomitant medications taken likewise failed to reveal any sign of harmful effect of the study treatment.

## Discussion

The safety analysis after implantation of three dose levels of the three-dimensional autologous chondrocyte implantation product ACT3D-CS and a 3-year follow-up shows that independently of dose, the product appears to be safe in the treatment of cartilage defects (4–10 cm^2^) of knee joints. No substantial differences were observed in the overall incidence of adverse events, in the numbers of patients with any adverse events, or the numbers of patients with treatment-related adverse events.

The adverse event ‘joint effusion’ was frequent in all dose groups and included one possibly and one probably treatment-related severe event. In the first 2 years of the study, one patient experienced two episodes of chondropathy, which were considered probably treatment-related; in the third year, two patients also experienced chondropathy, one considered unrelated and one unlikely to be related to the study treatment. An episode of arthralgia in the affected joint during the third year after treatment was considered to be probably treatment-related. Other safety analyses showed no unwanted effects of the study treatment.

In contrast to Carticel, an autologous cultured chondrocyte product that has been approved by the US Food and Drug Administration, the number of adverse events requiring surgical treatment, was very low in this 36-month follow-up (re-treatment of the treated lesion in the case of a patient with delamination and minor surgical treatment in another case for resection of hypertrophy, both considered probably related to the study treatment). After Carticel implantation, 294 spontaneous adverse event reports (497 adverse events) were submitted to the manufacturer and subsequently to the Food and Drug Administration in a 7-year period. Of the patients affected, 273 (93%) had a total of 389 surgical revisions, of which 187 (48%) involved subsequent cartilage procedures for the treatment of problems directly related to the graft [13].

Findings comparable to those of this investigation were observed in comparison with the safety data after characterised Chondrocyte Implantation, (ChondroCelect, TiGenix, Leuven, Belgium), the first cell-based therapy to be approved by the European Medicines Agency for the treatment of symptomatic isolated full-thickness cartilage defects of the femoral condyles. There, a total of 98% of patients experienced at least one treatment-emergent adverse event during the 60-month study period [14]. In the 36-month follow-up of the same study, 88% experienced at least one adverse event, with arthralgia being the most common adverse event that was treatment-related. The total number of treatment-related adverse events were reported by 70% of the patients, with only two events (deep vein thrombosis and tendinitis/tendinosis) classified as serious adverse events [7]. The number of serious adverse events was five, lower than in the present study; two were considered unlikely to be related to the procedure [7, 14] (hypersensitivity and ligament rupture), two as possibly related (deep vein thrombosis and arthralgia) and one as probably related (tendonitis).

The deviations from protocol that were most likely to have any influence upon the study’s safety results were the reduced dose administered to several patients in the high-dose group and the failure to obtain spheroids from two patients, so that these two did not undergo the implantation procedure. The reduced dose level could have biased the numerical results for the relative frequencies of inherently dose-related adverse events. The non-implantation of treatment in two patients must be borne in mind when the frequencies of adverse events are interpreted; however, its influence cannot have been great (e.g. an adverse event with an incidence of six patients will have had a relative frequency of 6/75 or 8.0% in the safety population and 6/73 or 8.2% in the population of patients who actually received spheroids). Apart from the dosing issue, which is not likely to have had any major effect upon the study results, compliance with the dosing regimen and with the rehabilitation measures was good. The patients’ medical history was unremarkable, and the dose groups were well balanced, particularly in respect of the distribution of defect size.

## Conclusions

The results of this 3-year safety analysis show that no dose relationship was detected and the treatment with chondrosphere® was generally well tolerated. No substantial differences in the incidence of any adverse events, or of patients with treatment-related adverse events, were observed.

## Abbreviations

ACI: Autologous chondrocyte implantation
ACT3D-CS: Autologous three-dimensional chondrocyte transplantation with chondrosphere®
ICRS-CRA: International Cartilage Repair Score, Cartilage Repair Assessment
KOOS: Knee Injury and Osteoarthritis Outcome Score
MRI: Magnetic resonance imaging
SOC: System organ class
